# Investigating health-related barriers to green space use, chronic health conditions and sociodemographic characteristics: a structural equation modelling approach

**DOI:** 10.1136/bmjph-2025-003077

**Published:** 2026-02-11

**Authors:** Hannah Burnett, Jonathan R Olsen, Sarah E Rodgers, Richard Mitchell

**Affiliations:** 1 Public Health, Policy and Systems, University of Liverpool, Liverpool, UK; 2 Institute for Social Science Research, The University of Queensland, Brisbane, Queensland, Australia; 3 MRC/CSO Social and Public Health Sciences Unit, University of Glasgow, Glasgow, UK

**Keywords:** Public Health, Cross-Sectional Studies, statistics and numerical data, Sociodemographic Factors

## Abstract

**Introduction:**

Using green space improves health and well-being. However, there are many barriers to green space use, including poor health. Evidence is lacking on how health-related barriers to green space differ by chronic health condition. This study investigates health-related barriers to green space use, chronic health conditions and sociodemographic characteristics.

**Methods:**

We assessed health-related barriers to green space use using Natural England’s People and Nature Survey. Data from 5 months of the nationally representative survey of English adults (aged 16+) were used (n=10 415), collected during November 2020–March 2021. To assess relationships between reporting of health-related barriers to using green space, an individual’s chronic health conditions and sociodemographic characteristics, structural equation modelling was used (n=201).

**Results:**

Respondents with progressive illnesses or physical disabilities had a higher likelihood of reporting multiple (four) health-related barriers as important compared with respondents with arthritis or diabetes (both had no barriers that they were more likely to report). For example, respondents with physical disabilities (32%) and progressive illnesses (31%) had an increased likelihood of reporting lack of disabled facilities as an important barrier to using green space compared with those with other conditions. Those with progressive illnesses (34%) had a particularly higher likelihood of reporting having no one to go with/help as an important barrier to using green space (p=0.001). Both physical health-related (eg, fatigue) and place-based (eg, poorly maintained sites) factors are barriers to green space use for individuals reporting a chronic condition.

**Conclusions:**

The results suggest that those with physical disabilities and progressive illnesses would benefit most from reducing place-based and support barriers, such as a lack of disabled facilities and no one to go with/help. By improving the suitability of green spaces using this evidence, barriers to green space would be reduced for all users, which may improve the quality of the space.

**WHAT IS ALREADY KNOWN ON THIS TOPIC**

- Green spaces benefit health and well-being. These benefits are not experienced equally, with inequalities widening.
- Poor health is one of the most commonly reported barriers to green space use.

**WHAT THIS STUDY ADDS**

- The results show that for those reporting health-related barriers, to increase their green space use would require individual-level mobility/health support and infrastructure improvements that provide disabled facilities.
- A ‘one size fits all’ approach will not work in mitigating barriers to green space use for those with chronic health conditions.

**HOW THIS STUDY MIGHT AFFECT RESEARCH, PRACTICE OR POLICY**

- The barriers to using greenspace vary by the type(s) of chronic condition, highlighting that condition-specific interventions are required and improving one aspect/barrier of a park may not provide equitable access for those with a range of chronic conditions.

## Introduction

 There is a wide range of evidence outlining the positive connections between green space and health, with the amount of literature exploring these associations increasing greatly over the past decade. The health benefits of green space reported in the existing literature are varied, including stress reduction, social cohesion, promotion of physical activity and reduced exposure to noise, air pollution and heat.^1^,^3^ These benefits can arise from use of green space, proximity and access to green space or residing in green areas. In this article, we are interested in actual visits to (or use of) green space.

The benefits of green space, however, are not experienced equally among population groups.^4 5^ Inequalities in use of green space are increasingly prevalent. These inequalities were also exacerbated during the COVID-19 pandemic, with the gap in green space visits between higher and lower social grade groups widening.^6 7^

Infrequent users of green space are more likely to be female, older, in poor health, of lower socioeconomic status and of ethnic minority status.^4^ Boyd *et al* found that being too busy at work and poor health were the most commonly reported reasons for infrequent use of green space in England. Additional barriers to green space use reported in the existing literature include a lack of interest, transport, weather and a lack of facilities, safety and cost.^5 8 9^

Poor health has been reported as a key barrier to use of green space.^410^,^12^ However, health-related barriers have either been explored using the broad term ‘poor health’, focused on barriers for people with specific health conditions (eg, dementia) with small sample sizes or focused on specific demographic groups or outcomes, such as older age groups and general physical activity. This indicates that surveying non-users of green space is challenging and highlights the need to explore differences in health-related barriers by chronic health conditions (eg, comparing barriers felt by those with respiratory conditions, physical disabilities and heart-related conditions in one analysis) and by sociodemographic characteristics to further understand if age, sex and ethnicity, for example, affect health-related barriers to using green spaces.

Individuals with chronic, or long-term, health conditions are more likely to be infrequent green space users than those without chronic health conditions.^4^ To increase their green space use for individuals with chronic health conditions, we first need to understand current barriers to using green space using nationally representative data. This could provide evidence for future interventions to improve green spaces for the groups that could benefit the most.

The aim of this study is to answer the following research questions.

- How do health-related barriers to using green space differ by (1) type of chronic health condition and (2) sociodemographic characteristics?

There will be a particular focus on exploring whether physical health-related barriers (ie, mobility and health) are more significant in influencing use of green space than place-based barriers (ie, poorly maintained site and lack of disabled facilities) for those with chronic health conditions.

## Materials and methods

### The people and nature survey (PANS)

Data from 5 months of the PANS were used, collected during November 2020–March 2021.^13^ The PANS was created by Natural England in 2020 to gather data from online respondents aged 16 and over, asking about enjoyment, access and attitudes towards the natural environment in England.^14 15^ The PANS survey and its predecessor, the Monitor of Engagement with the Natural Environment survey, have been previously used to explore non-users of green spaces and reasons for non-use, as well as the impact of the COVID-19 pandemic on use of green spaces, nature connectedness and well-being.^4 16 17^

The PANS survey is ongoing with data collected monthly through Kantar, a data and evidence-based agency that runs multiple online survey panels. An English subset of Kantar’s Profiles online panel is invited to participate in PANS.^14^ The Kantar panel uses a diverse set of recruitment sources and methods, including opt-in email, coregistration and e-newsletter campaigns. Weights were included in the dataset to enable the subsequent analysis to accurately represent the national population of England.^14^

#### Health-related barriers to green space use

**Figure 1:**
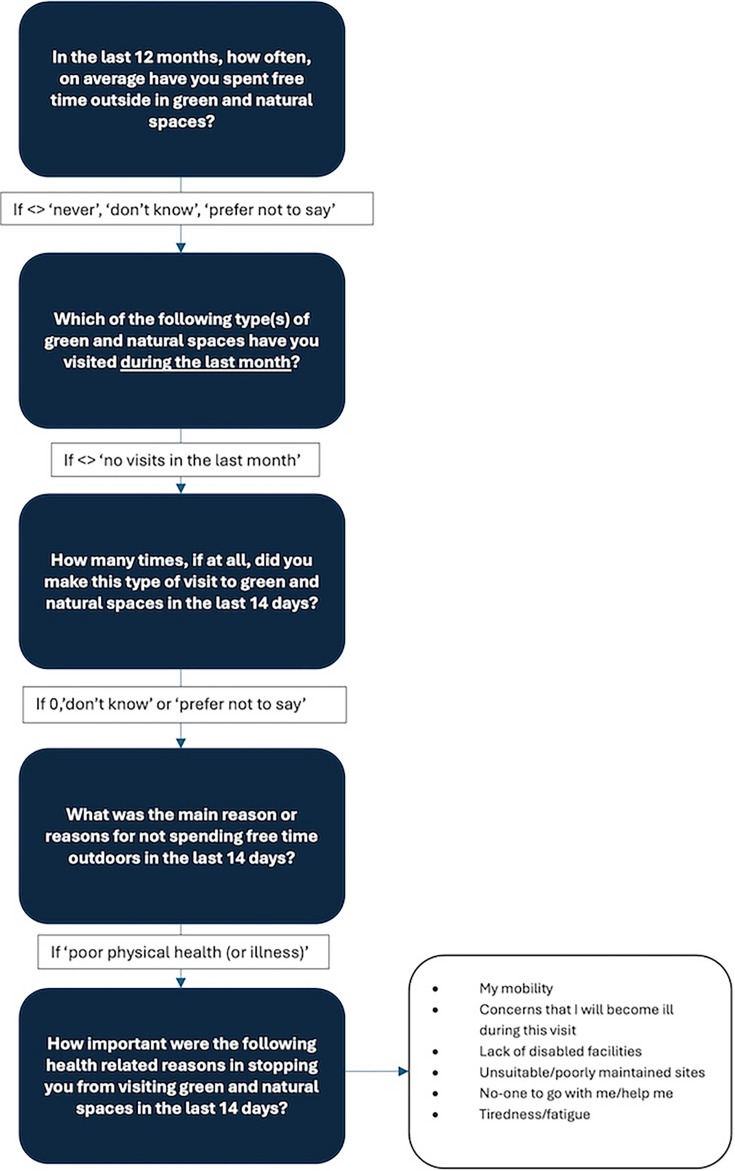
Flowchart presenting the survey routing for the health-related barrier questions. The health-related barriers were then rated from 1 (not at all important) to 5 (very important).

The PANS has a set of modules, which are asked every month, with certain modules asked of a randomly selected subsample of individuals. The PANS asked respondents whether they had visited green space in the last 14 days; for this study, we only analysed data for those who reported they had not visited green space in the last 14 days. Figure 1 highlights the questions asked in the survey and the survey design flow specific to questions regarding use of greenspace and barriers to greenspace use.

To explore whether physical health-related barriers are more significant in influencing use of green space than place-based barriers, these health-related barriers were categorised into physical health-related barriers (my mobility, concerns that I will become ill during this visit and tiredness/fatigue) and place-based barriers (lack of disabled facilities and unsuitable/poorly maintained sites), with no one to go with me/help me analysed as an individual support barrier.

#### Chronic health conditions

We funded a new question to ask respondents about the type of chronic health condition they had, if any. This question was created by adapting questions on health status and conditions from existing surveys, such as the Scottish Household Survey and Scottish Health Survey. The new question was included during the period November 2020–March 2021. The chronic health conditions were included in PANS as a multiple-choice list selected by the respondent if they had reported having one or more physical or mental health conditions or illnesses lasting or expecting to last for 12 months or more (response of ‘yes’ or ‘no’). The chronic health conditions included in this analysis were as follows.

- Arthritis or joint-related conditions.
- Respiratory or breathing problems, for example, asthma.
- Diabetes.
- Heart, blood pressure or circulation problems.
- Another physical disability.
- Another progressive disability, illness or health problem (ie, that can get worse over time, such as dementia or Parkinson’s disease)

Respondents could also choose ‘prefer not to say’, ‘don’t know’ or ‘other (please specify)’.

#### Sociodemographic variables

Sociodemographic variables were included in the analysis.

- Sex (binary)—Male (0), Female (1).
- Age (continuous)—exact age of respondents (16+years).
- Income (categorical)—£0–19 999 (1), £20 000–39 999 (2), £40 000–59 999 (3), £60 000–99 999 (4) and £100 000+ (5).
- Ethnicity (binary)—White (0), Black, Asian, and Minority Ethnic (BAME) (1).

### Data analysis

Descriptive statistics (counts and percentages) were used to explore the number of respondents reporting a chronic health condition and if these counts differed by sociodemographic characteristics.

To assess relationships between reporting of health-related barriers to using green space, an individual’s chronic health conditions and sociodemographic characteristics, structural equation modelling (SEM) was used. The method of SEM enables the evaluation of relationships among latent variables; variables that cannot be directly observed but can be derived from other variables, by combining the strengths of factor analysis and multiple regression into a single model that can then be tested statistically.^18^ SEM analysis was required to explore whether physical health-related barriers (ie, mobility and health) are more significant in influencing use of green space than place-based barriers (ie, poorly maintained site and lack of disabled facilities) for those with chronic health conditions while adjusting for age, sex, income and ethnicity.

**Figure 2:**
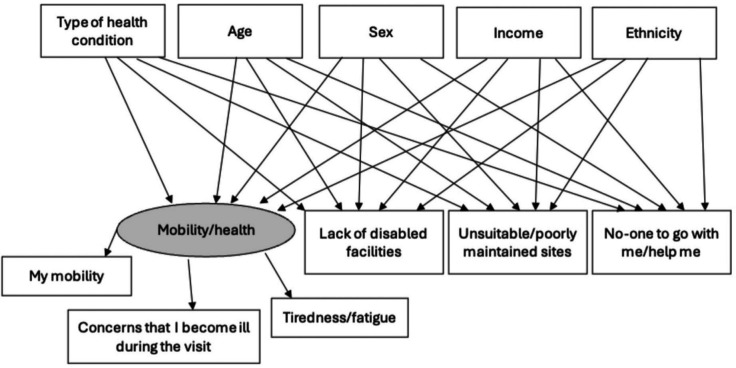
The initial plan for the SEMs focusing on health-related barriers to green space use. The type of health condition and sociodemographic variables at the top of the figure are the independent (predictor) variables, while the health-related barriers at the bottom of the figure are the dependent variables. The grey-shaded, circular line around ‘mobility/health’ signifies that this is a latent variable, while the variables in rectangular boxes are observed variables from PANS. PANS, people and nature survey; SEM, structural equation modelling.

The SEMs, based on figure 2, were run in R using packages lavaan (V.0.6–9), semptools (V.0.2.9.3) and lavaan.survey (V.1.1.3.1). The variables included in the SEM were categorical (health conditions, health-related barriers, sex, income and ethnicity), except for age. An SEM was created for each type of health condition to explore associations between the predictor variables (type of health condition and sociodemographic variables) and the outcome variables (health-related barriers).

To explore whether physical health-related barriers (ie, mobility and health) were more significant than place-based barriers (ie, poorly maintained site and lack of disabled facilities) as reasons for not visiting green space for those with chronic health conditions, the latent variable ‘mobility/health’ was created by combining three observed variables: ‘my mobility’, ‘concerns that I become ill during the visit’ and ‘tiredness/fatigue’. The observed variables adequately measured the latent variable.^19 20^ More than two observed variables are required to create a latent variable; therefore, a latent variable for place-based barriers could not be created using these data.^21^

## Results

The results presented in table 1 suggest differences in reporting health conditions by sociodemographic characteristics.

**Table 1:** Weighted count and percentage of respondents reporting a chronic health condition by sex (binary), age (categorised for descriptive analysis), ethnicity (binary) and income (categorical)

	Count reporting chronic condition	Percentage reporting chronic condition
Sex		
Male	1974	39
Female	2171	41
Age		
16–24	454	33
25–39	974	36
40–54	933	39
55–64	654	42
65+	1142	48
Ethnicity		
BAME	453	32
White	3634	42
Income		
£0–19 999	1348	50
£20 000–39 999	1564	42
£40 000–59 999	700	33
£60 000+	521	29

**Figure 3:**
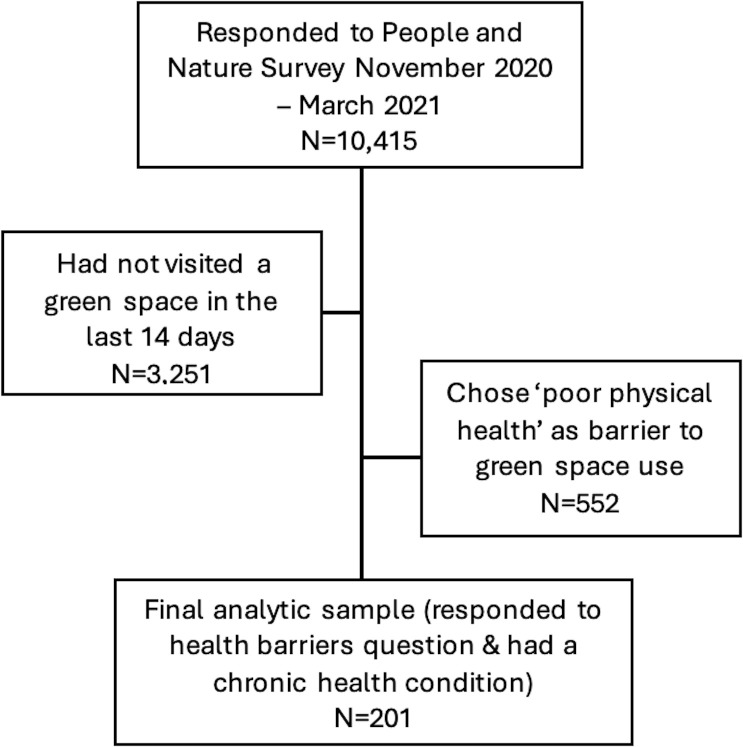
STROBE diagram of analytical sample with weighted counts. STROBE, Strengthening the Reporting of Observational Studies in Epidemiology.

A minimum sample size of 100 is recommended for SEM, although this depends on the complexity of the model.^22^ Overall, the final sample size available was 201, which reflects the number of respondents who reported a chronic health condition (excluding those reporting mental health conditions), chose ‘poor physical health’ as a general barrier to green space use and responded to the health-related barrier question (with do not know, prefer not to say and N/A responses being excluded) (figure 3). Therefore, respondents reporting a mental health condition were excluded because of the focus on poor physical health and the option to respond ‘poor mental health and well-being’ to the question regarding general barriers to using green space.

The factor loadings were acceptable for each model, with good model fit (online supplemental table S1). A post hoc power analysis was calculated in R using the semPower package (V.2.1.3). With the sample size of 201 within the SEMs, the result of the power analysis was >0.99 and, therefore, ‘good’ at >80%.^23^

### Research question: how does the reporting of health-related barriers to using green space differ by type of health condition?

Differences in reporting health-related barriers to green space use depended on the type of health condition reported (table 2). For example, respondents with progressive illnesses were the most likely to report all of the health-related barriers as important, compared with respondents with other chronic health conditions. In contrast, respondents with arthritis were not statistically significantly more likely to report any of the barriers as important. All SEM figures are available in the online supplemental figures S2–S6.

**Table 2:** SEM regression results for the types of health condition

	Mobility/health			Lack of disabled facilities		
	P value	Std estimate (95% CI)	SE	P value	Std estimate (95% CI)	SE
Arthritis	0.307	0.11 (−0.15 to 0.47)	0.156	0.211	0.12 (−0.14 to 0.63)	0.196
Respiratory	0.026*	0.28 (0.05 to 0.82)	0.196	0.224	0.12 (−0.16 to 0.69)	0.216
Diabetes	0.253	0.13 (−0.17 to 0.63)	0.203	0.075*	0.19 (−0.05 to 0.99)	0.264
Heart/blood pressure/circulatory	0.002**	0.34 (0.19 to 0.87)	0.174	0.007**	0.28 (0.16 to 1.02)	0.218
Physical disability	0.01**	0.28 (0.10 to 0.74)	0.163	0.001***	0.32 (0.30 to 1.01)	0.201
Progressive illness	0.013**	0.27 (0.07 to 0.81)	0.180	0.008**	0.31 (0.26 to 1.19)	0.212

	Unsuitable/poorly maintained sites			No one to go with/help me		
	P value	Std estimate (95% CI)	SE	P value	Std estimate (95% CI)	SE
Arthritis	0.975	−0.00 (−0.39 to 0.37)	0.195	0.353	0.09 (−0.20 to 0.57)	0.196
Respiratory	0.45	0.08 (−0.27 to 0.61)	0.224	0.139	0.15 (−0.11 to 0.75)	0.219
Diabetes	0.547	0.06 (−0.34 to 0.64)	0.251	0.314	0.10 (−0.23 to 0.71)	0.239
Heart/blood pressure/circulatory	0.057*	0.20 (−0.01 to 0.84)	0.219	0.367	0.09 (−0.22 to 0.60)	0.211
Physical disability	0.053*	0.18 (−0.01 to 0.74)	0.190	0.047**	0.17 (0.01 to 0.73)	0.185
Progressive illness	<0.001***	0.33 (0.36 to 1.27)	0.204	0.001***	0.34 (0.38 to 1.30)	0.215

*p<0.1; **p<0.05 and ***p<0.001.

SEM, structural equation modelling.

The exact standardised estimates, CIs and p value results are presented in table 2, with the significant associations (p<0.05) highlighted by asterisks. The p values that were >0.05 but <0.1 are also emphasised by asterisks. The threshold value of p<0.05 as significant is arbitrary, and the dichotomous ‘significant’ and ‘non-significant’ categories mean that information can be lost.^24^ Therefore, we have included a less stringent threshold of p<0.1 to ensure that all possible associations are explored. The results with p values <0.1 may have a weak association, but one potentially worth exploring further.

For the direct effects, standardised estimates (along with their SE) are reported with 95% CIs and p values. To interpret results, we describe standardised estimates with a value from 0 to 0.20 as small, from 0.20 to 0.40 as medium and greater than 0.40 as large.^25^ For clarity of presentation of the results, in the text, we only report the standardised estimate size.

All standardised estimates were either small or medium value (0–0.40), with none being greater than 0.4. The CIs were generally wide across the results, despite the size of the p values, which further suggest small effects.

#### Mobility/health

The majority of health conditions were associated with reporting of ‘mobility/health’ as an important barrier, with p values<0.05. The standardised estimates show that respondents with respiratory conditions (28%), heart/blood pressure/circulatory conditions (34%), physical disabilities (28%) and progressive illnesses (27%) all had an increased likelihood for their mobility/health being an important barrier to green space use. Each of these results had wide CIs, such as respiratory conditions (Std estimate: 0.28, 95% CI 0.05 to 0.82).

#### Lack of disabled facilities

Respondents with heart/blood pressure/circulatory conditions (28%), physical disabilities (32%) and progressive illnesses (31%) had an increased likelihood of reporting a lack of disabled facilities as an important barrier to using green space (table 2). Respondents with diabetes had a 19% increased likelihood of reporting this barrier, but with a p value <0.1 and very wide CIs.

#### Unsuitable/poorly maintained sites

Respondents with progressive illnesses had a strong association with reporting unsuitable/poorly maintained sites as an important barrier (p<0.001) to using green space. These respondents had a 33% increased likelihood of reporting this barrier as important. Respondents with physical disabilities (18%) and heart/blood pressure/circulatory conditions (20%) had weaker increased likelihoods of reporting unsuitable/poorly maintained sites as an important barrier, with p values closer to 0.05 and wide CIs.

#### No one to go with/help me

For this health-related barrier, there were associations for respondents with two types of health conditions, with p values <0.05. Respondents with physical disabilities (17%) and progressive illnesses (34%) had higher likelihoods of reporting having no one to go with/help as an important barrier to using green space.

### Research question: how does the reporting of health-related barriers to using green space differ by sociodemographic characteristics?

There were no differences in the reporting of physical health-related barriers by sex, age, income and ethnicity, when adjusted for each type of health condition (online supplemental table S7). The only p value <0.1 was for the age variable in the SEM for respiratory conditions (p=0.052), with a 22% lower likelihood of reporting mobility/health as important as age increased.

## Discussion

This study explores the health-related barriers to green space use in England for those who had reported not using green space over the past 14 days. Our findings suggest that there are differences in the reporting of health-related barriers to using green space by type of chronic health condition. The larger relative effects for both physical health-related and place-based barriers suggest that these are equally significant barriers to green space use, particularly for respondents with physical disabilities, progressive illnesses and circulatory conditions. For example, respondents with progressive illnesses had a higher likelihood of reporting all barriers as important compared with respondents with arthritis or diabetes who had no statistically significant results. There were no barriers that were consistently reported across all chronic health conditions.

Existing research corroborates to some extent with the finding in our study that mobility/health was the health-related barrier most likely reported by people with multiple types of health conditions (all except arthritis and diabetes). For example, a study found that respondents reported internal barriers (such as tiredness) as more constraining than environmental and social barriers (such as family not being encouraging and too few places to exercise) regarding exercising, regardless of a respondent’s disability type.^26^

The results suggest that those with physical disabilities and progressive illnesses would benefit most from reducing health-related barriers, such as a lack of disabled facilities and no one to go with/help. The findings could be used in the design of social programmes taking place within green spaces, such as parks, to ensure accessibility for those with physical disabilities and progressive illnesses. For example, ensuring access to suitable toilet facilities and organising peer support walking and wheeling groups. A successful example of this is evidenced by the Scottish Charity Paths for all, who run Dementia Friendly Health Walks that are accessible and inclusive for those living with dementia and their carers.^27^ The changes made, and interventions created, to reduce barriers for certain groups can make the spaces more accessible and improve quality for all.

We found relatively weaker associations between sociodemographic characteristics and health-related barriers, based on both the p value (with only one p value <0.1 and none <0.05). This suggests that the health conditions were the key drivers behind these health-related barriers. There was variation in reporting health conditions by sociodemographic variables in the descriptive statistics, including income, which could exacerbate some of the variations in green space use. For example, some of the relationships between income and no/low use of green space could be mediated through poor health.^28^ Tackling the health-related barriers to green space use could have the potential to reduce the existing inequalities in green space use, with the biggest proportional impact for the lower income group who have a higher proportion of respondents reporting a chronic health condition. We suggest further study using a larger dataset to explore the relationship among income, health and barriers to green space use.

Older adults, those in lower socioeconomic status groups, and those with a long-term illness/disability were more likely to report poor health as a barrier to visiting green space frequently.^4^ Similarly, a study from the USA found that with each additional year of age, the odds of reporting poor health as a reason for not visiting National Forests increased by a factor of 1.05.^29^ These findings suggest that differences exist between sociodemographic groups in the reporting of health barriers to green space, particularly for people at different ages. There may be widening inequalities as people age. These findings encourage intersectionality in green space research, examining the interacting influences of multiple axes of inequality (such as socioeconomic status, gender and ethnicity).^30^

Implementing interventions to remove health-related barriers to green space use is becoming more important, with the reporting of chronic conditions within younger populations in the UK increasing. The starkest rise has been among 16–34 year olds, where the rate of work-limiting health conditions has doubled over the past 10 years.^31^ More generally, the number of people living with major illness in England is projected to increase by more than a third, reaching almost 1 in 5 by 2040.^32^ Therefore, the population reporting health-related barriers to using green space will increase, and implementing the interventions to remove these barriers becomes even more important.

### Strengths and limitations

This study had several limitations. First, the data being collected from November 2020 to March 2021 limits the generalisability to other times of the year due to the potential impact of seasonality on green space use and barriers. Despite this, the analysis within the sample is robust due to data being collected during the same period. This data collection period was also during the COVID-19 pandemic, with perceptions of green space and barriers likely to differ during the winter lockdowns and significant change to everyday life. Further, the cross-sectional design of the study means that causal inferences cannot be drawn from the associations presented. The health conditions were self-reported by survey respondents rather than gathered from administrative data, which create further limitations. For example, a study comparing self-reported and biomedical data on hypertension and diabetes found that self-reported health led to an underestimation of the prevalence of hypertension and diabetes.^33^ A further limitation was using only two categories for ethnicity, meaning that in-depth information on differences in green space use and barriers to use by ethnicity was lost during the analysis. The statistical analysis evaluates whether each health condition–barrier association differs from zero, resulting in descriptive cross-condition comparisons. Between-condition contrasts should be focused on in future analyses to test whether associations differ between each condition.

Despite these limitations, this study contributes novel and original findings by focusing on barriers to green space use and how these differ by health condition which, to the authors’ knowledge, had not been explored to this extent in a UK context. The non-user population, particularly those with health-related barriers, is hard to reach compared with research focusing on the benefits of green space use, when participants are identified while using a park or other type of green space. By focusing on poor health as a barrier, we were able to distinguish between multiple types of health conditions, which contribute new data and findings to the research field seeking to understand why people do or do not use green spaces. The weightings added to the PANS allowed for nationally representative results, strengthening the study and reducing risk of selection bias. There was also robust analysis undertaken using SEM to explore multiple factors contributing to infrequent/non-use of green space.

## Conclusion

Despite the positive impact that green space can provide for health and well-being, there are barriers that restrict many individuals from using green spaces and the subsequent benefits, which vary by type of chronic health condition. The findings emphasise that a ‘one size fits all’ approach will not work in mitigating barriers to green space use for those with chronic health conditions. This analysis of health-related barriers suggests that both physical health-related (eg, fatigue) and place-based (eg, poorly maintained sites) factors are barriers to green space use for individuals reporting a chronic condition. The results suggest that those with physical disabilities and progressive illnesses would benefit most from reducing health-related barriers, such as a lack of disabled facilities and no one to go with/help.

The barriers to using greenspace vary by the type of chronic health conditions, highlighting that condition-specific interventions are required to provide equitable access for those with a range of chronic conditions.

## Supplementary material

10.1136/bmjph-2025-003077 online supplemental file 1

## Data Availability

Data are available in a public, open access repository.
