# Supplementary material for: Investigating health-related barriers to green space use, chronic health conditions and sociodemographic characteristics: a structural equation modelling approach
Source: BMJ Public Health. 2026 Feb 11;4(1):e003077. doi: 10.1136/bmjph-2025-003077 (PMC12911733; doi:10.1136/bmjph-2025-003077)
Supplement: online supplemental file 1 [file bmjph-4-1-s001.docx]

**Supplementary Material**

Table S1: Goodness of fit for each model, with the definition of good fit included.

| **SEMs by health condition** | ***RMSEA*** | ***SRMR*** | ***CFI*** | ***P-value (Chi-square)*** |
| --- | --- | --- | --- | --- |
| ***Good fit*** | ***<0.06*** | ***<0.06*** | ***>0.90*** | ***>0.05*** |
| ***Arthritis*** | <0.001 | 0.035 | 1 | 0.604 |
| ***Respiratory conditions*** | <0.001 | 0.036 | 1 | 0.581 |
| ***Diabetes*** | <0.001 | 0.035 | 1 | 0.67 |
| ***Heart/blood pressure/circulatory conditions*** | <0.001 | 0.039 | 1 | 0.517 |
| ***Physical disabilities*** | <0.001 | 0.037 | 1 | 0.574 |
| ***Progressive illnesses*** | <0.001 | 0.037 | 1 | 0.704 |


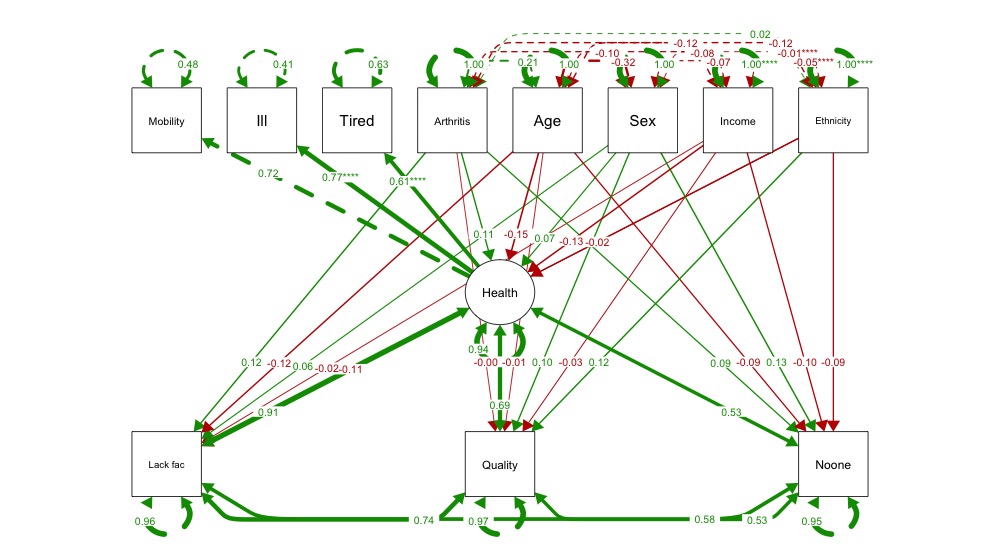


Maint

Figure S2: SEM for arthritis, socio-demographic variables, and health barriers – showing standardised estimates, *p<0.1 **P<0.05 ***p<0.01.


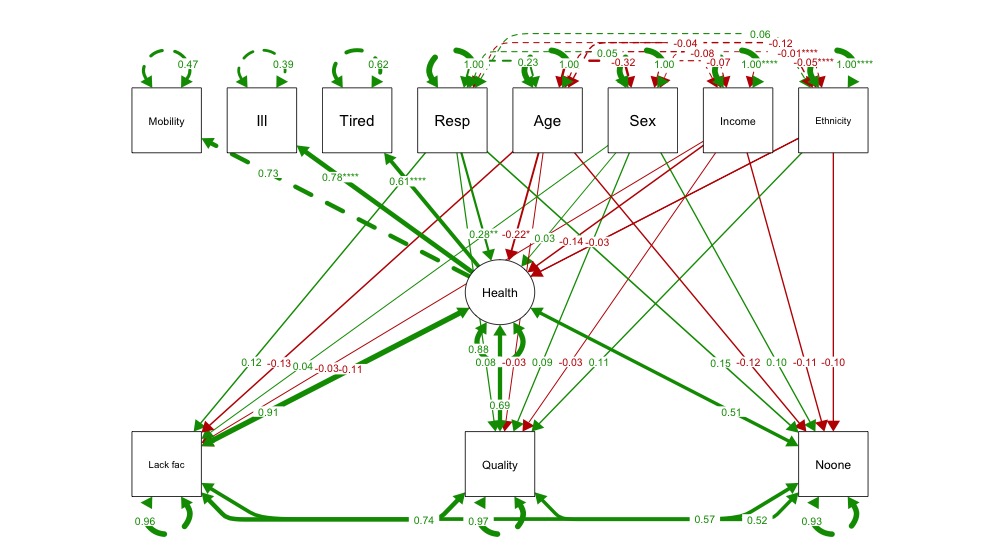


Maint

Figure S3: SEM for respiratory conditions, socio-demographic variables, and health barriers – showing standardised estimates, *p<0.1 **P<0.05 ***p<0.01, ****p<0.001.

Figure S4: SEM for diabetes, socio-demographic variables, and health barriers – showing standardised estimates, *p<0.1 **P<0.05 ***p<0.01, ****p<0.001.


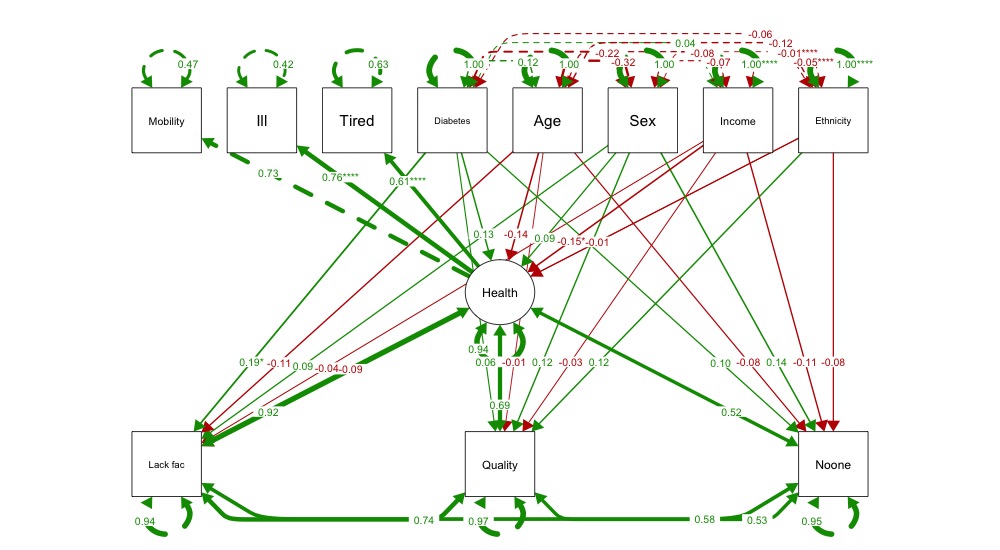


Maint


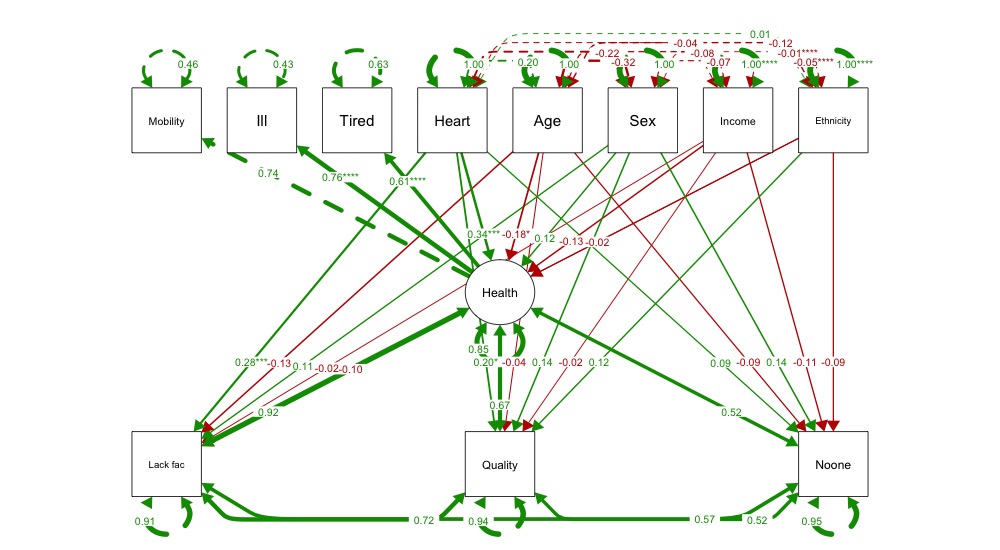


Maint

Figure S5: SEM for heart/blood pressure/circulatory conditions, socio-demographic variables, and health barriers – showing standardised estimates, *p<0.1 **P<0.05 ***p<0.01, ****p<0.001.


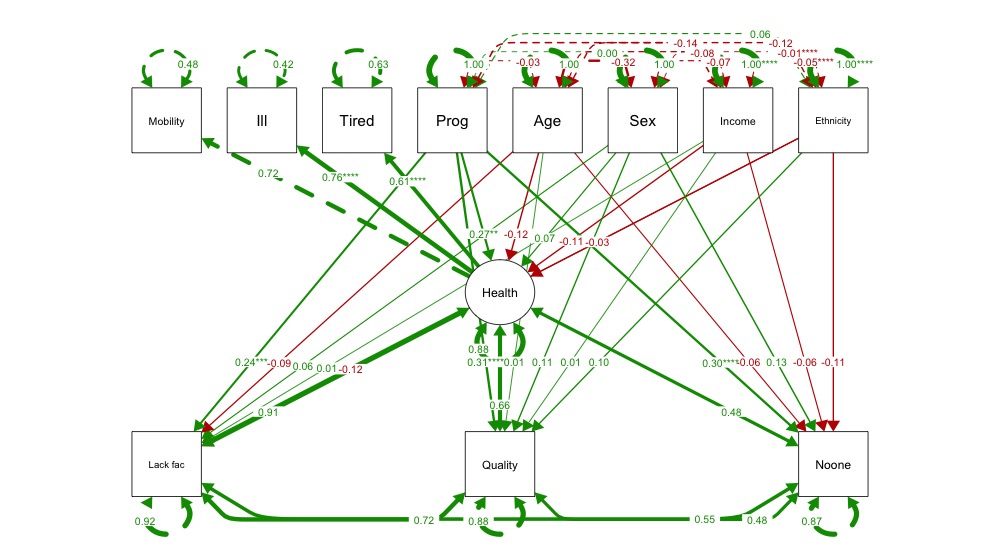


Maint

Figure S6: SEM for progressive illnesses, socio-demographic variables, and health barriers – showing standardised estimates, *p<0.1 **P<0.05 ***p<0.01, ****p<0.001.

|  | Mobility/health | | Lack of disabled facilities | | Unsuitable / poorly maintained sites | | No-one to go with/help me | |
| --- | --- | --- | --- | --- | --- | --- | --- | --- |
|  | P-value | Std Estimate | P-value | Std Estimate | P-value | Std Estimate | P-value | Std Estimate |
| *Arthritis* |  |  |  |  |  |  |  |  |
| Age | 0.204 | -0.15 | 0.221 | -0.122 | 0.937 | -0.008 | 0.363 | -0.091 |
| Sex | 0.537 | 0.07 | 0.576 | 0.058 | 0.298 | 0.104 | 0.184 | 0.126 |
| Income | 0.138 | -0.132 | 0.831 | -0.019 | 0.712 | -0.032 | 0.249 | -0.098 |
| Ethnicity | 0.773 | -0.023 | 0.17 | -0.108 | 0.176 | 0.12 | 0.297 | -0.092 |
| *Respiratory* |  |  |  |  |  |  |  |  |
| Age | 0.052 | -0.215 | 0.189 | -0.133 | 0.743 | -0.033 | 0.229 | -0.12 |
| Sex | 0.806 | 0.029 | 0.734 | 0.036 | 0.373 | 0.092 | 0.304 | 0.101 |
| Income | 0.116 | -0.137 | 0.717 | -0.033 | 0.721 | -0.032 | 0.204 | -0.11 |
| Ethnicity | 0.67 | -0.033 | 0.141 | -0.115 | 0.203 | 0.112 | 0.222 | -0.103 |
| *Diabetes* |  |  |  |  |  |  |  |  |
| Age | 0.21 | -0.139 | 0.264 | -0.109 | 0.9 | -0.012 | 0.402 | -0.08 |
| Sex | 0.444 | 0.094 | 0.387 | 0.093 | 0.268 | 0.116 | 0.143 | 0.142 |
| Income | 0.1 | -0.146 | 0.653 | -0.039 | 0.703 | -0.033 | 0.174 | -0.112 |
| Ethnicity | 0.935 | -0.006 | 0.228 | -0.093 | 0.158 | 0.124 | 0.339 | -0.084 |
| *Heart/blood pressure/circulatory* | | |  |  |  |  |  |  |
| Age | 0.093 | -0.179 | 0.167 | -0.134 | 0.692 | -0.038 | 0.399 | -0.086 |
| Sex | 0.271 | 0.123 | 0.303 | 0.106 | 0.194 | 0.137 | 0.148 | 0.139 |
| Income | 0.134 | -0.129 | 0.781 | -0.024 | 0.795 | -0.022 | 0.205 | -0.106 |
| Ethnicity | 0.747 | -0.023 | 0.155 | -0.104 | 0.186 | 0.116 | 0.3 | -0.09 |
| *Physical disability* | |  |  |  |  |  |  |  |
| Age | 0.38 | -0.096 | 0.594 | -0.05 | 0.878 | 0.015 | 0.584 | -0.051 |
| Sex | 0.485 | 0.08 | 0.49 | 0.068 | 0.264 | 0.113 | 0.155 | 0.132 |
| Income | 0.101 | -0.14 | 0.772 | -0.025 | 0.751 | -0.027 | 0.187 | -0.107 |
| Ethnicity | 0.788 | -0.022 | 0.188 | -0.106 | 0.179 | 0.123 | 0.299 | -0.089 |
| *Progressive illness* | |  |  |  |  |  |  |  |
| Age | 0.274 | -0.119 | 0.36 | -0.087 | 0.94 | 0.007 | 0.503 | -0.058 |
| Sex | 0.537 | 0.071 | 0.601 | 0.055 | 0.268 | 0.107 | 0.157 | 0.132 |
| Income | 0.247 | -0.105 | 0.931 | 0.008 | 0.868 | 0.014 | 0.426 | -0.062 |
| Ethnicity | 0.751 | -0.026 | 0.117 | -0.118 | 0.198 | 0.102 | 0.195 | -0.109 |

Table S7: SEM results for sociodemographic variables by health condition/model.
